# Novel Signal Noise Reduction Method through Cluster Analysis, Applied to Photoplethysmography

**DOI:** 10.1155/2018/6812404

**Published:** 2018-01-29

**Authors:** William Waugh, John Allen, James Wightman, Andrew J. Sims, Thomas A. W. Beale

**Affiliations:** ^1^Institute of Cellular Medicine, Newcastle University, Newcastle upon Tyne NE2 4HH, UK; ^2^Northern Medical Physics and Clinical Engineering, Newcastle upon Tyne NHS Foundation Trust, Newcastle upon Tyne NE7 7DN, UK

## Abstract

Physiological signals can often become contaminated by noise from a variety of origins. In this paper, an algorithm is described for the reduction of sporadic noise from a continuous periodic signal. The design can be used where a sample of a periodic signal is required, for example, when an average pulse is needed for pulse wave analysis and characterization. The algorithm is based on cluster analysis for selecting similar repetitions or pulses from a periodic single. This method selects individual pulses without noise, returns a clean pulse signal, and terminates when a sufficiently clean and representative signal is received. The algorithm is designed to be sufficiently compact to be implemented on a microcontroller embedded within a medical device. It has been validated through the removal of noise from an exemplar photoplethysmography (PPG) signal, showing increasing benefit as the noise contamination of the signal increases. The algorithm design is generalised to be applicable for a wide range of physiological (physical) signals.

## 1. Introduction

Signal quality or signal-to-noise ratio requires consideration in almost all signal measurements. This is especially true in physiological measurements where the signals tend to be small and prone to measurement artefacts and the noise is often difficult to control. In this paper, a novel cluster analysis method is described to reduce the influence of noise on photoplethysmography (PPG) signals. PPG is an optical measurement technique that can be used to detect blood volume changes in the microvascular bed of tissue [1]. The peripheral pulse, as measured by PPG, is often used in the assessment of health and disease and can provide important valuable information about the cardiovascular system [2–5]. Our research group is evaluating PPG for the diagnosis of peripheral arterial disease in a primary care situation using a fully automated diagnostic device [6]. The clinical utility of such a device relies on its ability to identify and eliminate noise from PPG signals.

Noise minimisation starts with removing the source of the noise; this can be through electrical isolation or, for example, by keeping the subject relaxed and still during measurements to eliminate muscle and movement artefact. There is also inherent noise produced through the amplification of small signals; however modern physiological amplifiers and analogue-to-digital converters tend to minimise this for all but the smallest input signals. When the sources of the noise have been reduced as far as possible, various active noise reduction techniques can be used. The most common kind of noise minimisation is filtering [7, 8] that can be used to reduce any noise frequencies that do not overlap the signal frequencies. More sophisticated methods such as wavelet denoising [9] can be employed where filtering is insufficient. Physiological signals, in particular ECG and PPG, have been the focus of noise reduction using a signal quality index, whereby each pulse has attributed a signal quality, which is then used to assess the validity of that pulse [10–13].

Cluster analysis is a method of arranging features into groups such that those with similar characteristics lie within a single group. Cluster analysis is common in data analysis and there are many algorithms [14]. In this paper, we have applied a simple cluster analysis to remove noise from a physiological PPG signal. This signal is periodic and the disease diagnosis is performed from a representative sample pulse. Therefore, characteristics of individual pulses are not needed but rather characteristics from a representative single pulse (be this a selected good pulse or an average of pulses). In order to output a representative pulse, a trace with many pulses is recorded and the average pulse from these calculated. This method performs well when noise contamination is low, for example, when recorded in established physiological measurement settings and by trained researchers studying PPG. However, moving diagnostic devices into real-world clinical settings to provide a robust and automated assessment can be challenging. For example, patients may not stay still during the recording and the device must be designed to return a valid clinical result with any reasonable expected clinical setting and level of staff training. When significant measurement noise is present, this noise can dominate the average pulse such that this is no longer a true representation of the subject's PPG pulse. This paper describes an algorithm using cluster analysis to select a subset of pulses to return a representative pulse returned for subsequent diagnosis.

## 2. Method

The algorithm was developed using Matlab® version 2016b; the photoplethysmography and electrocardiogram signals were measured using a multichannel PPG and ECG recorder as used in a clinical study [15]. Prerecorded signals from normal subjects were used as an input to the algorithm. A variety of finger and/or toe pulse signals with noise implemented through on-purpose patient movement were used to train the algorithm. The algorithm was developed to run in real time such that the signal capture could be terminated when sufficient signal has been received. For a high-quality signal, this can result in a shorter recording time. For signals with a small signal-to-noise ratio, this allows the device to collect sufficient data such that a diagnosis is possible, up to a time-out limit.

### 2.1. Algorithm Development

The design requirements for the algorithm were as follows:

- Compute on a continuous digital data stream, with a minimal signal delay (pseudo-real-time).
- Remove low-frequency noise (DC drift).
- Remove high-frequency noise.
- Remove sporadic mixed-frequency noise.
- Terminate when sufficient “good” pulses are recorded.

The algorithm was developed to reduce noise from a PPG signal. This signal has a periodic frequency equal to the heart rate of the subject. The signal structure mainly exists in the low-frequency domain, with the desirable frequencies for analysis lying between 0.15 Hz and 20 Hz.

The algorithm's steps are shown in Figure 2. The algorithm can be divided into three sections: initial filtering and slicing of the data, pulse clustering, and termination.

### 2.2. Initial Filtering and Slicing Stage

The incoming PPG signal is subject to a digital bandpass filter to remove unwanted noise and signal drift. This is implemented through a low-pass filter and a high-pass filter, designed to minimise both the signal distortion and the signal phase delay. A minimal delay is imperative for any device where a live trace is shown, especially where operator feedback is a possibility (e.g., adjustment of the sensor at the measurement site). Any substantial delay can render such operator feedback confusing and nonintuitive. The options for digital filters fall into two main categories: FIR (Finite Impulse Response) and IIR (Infinite Impulse Response) [17]. Although symmetric FIR filters have the advantage that they have linear phase and are always stable, they have substantial delays when designed with low cut-off frequencies. IIR filters generally have a nonlinear phase response and therefore cause a frequency-related signal delay; however, they can be faster than an FIR filter.

**Figure 1:**
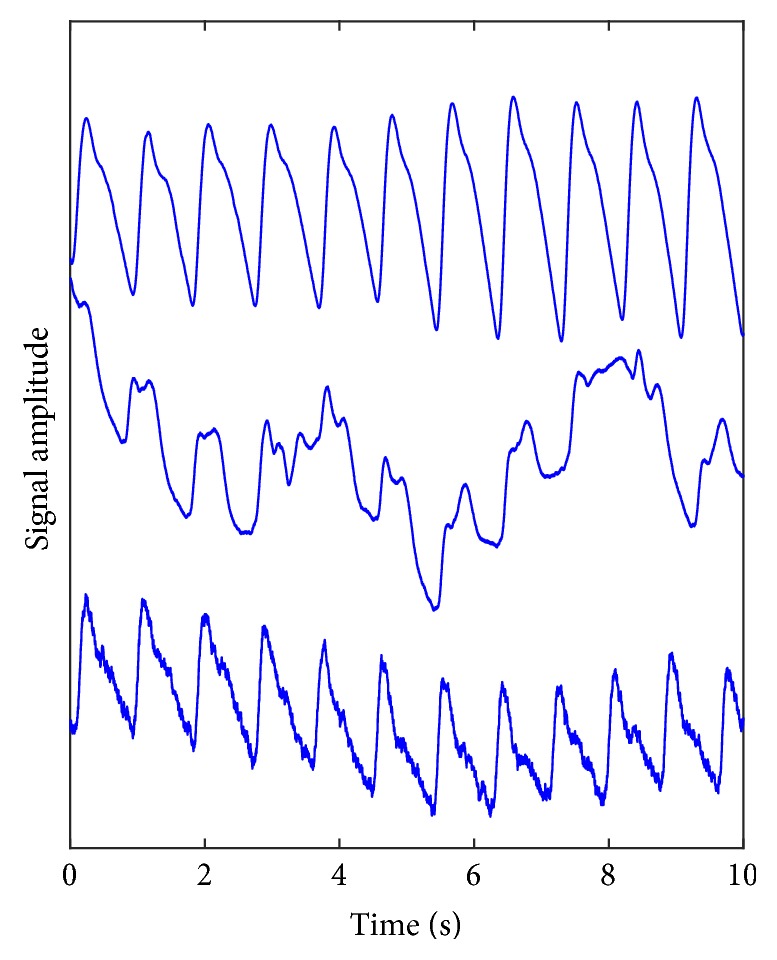
Three recorded photoplethysmography (PPG) traces measured from the great toe site. Upper: a clean PPG signal; middle: a signal dominated by low-frequency noise, usually caused by movement of the subject's limb; lower: a PPG trace contaminated with high-frequency noise, typical of electrical interference. Typically, these noise features can appear intermittently within a recording made over a measurement period of 1-2 minutes.

**Figure 2:**
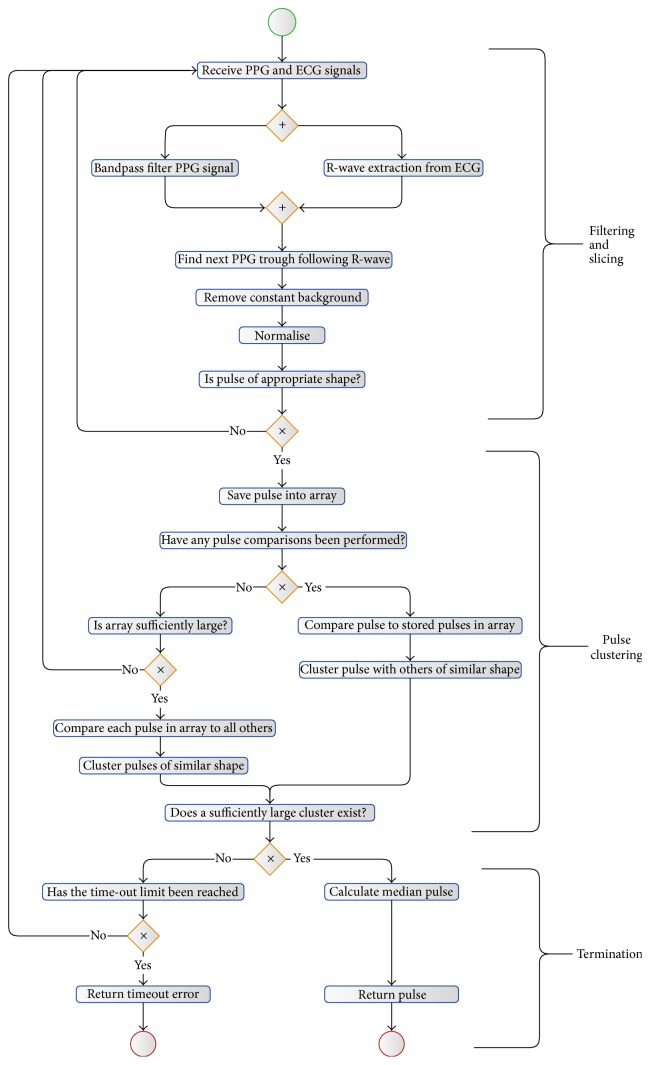
Algorithm's steps represented as a flow diagram, utilising BPMN Notation [16].

The information in the PPG signal lies in the low-frequency range (below ~20 Hz); however, the signal is often contaminated by high-frequency noise (Figure 1). This is often due to measurements in an electrically noisy environment or optical pick-up from external lighting sources.

**Figure 3:**
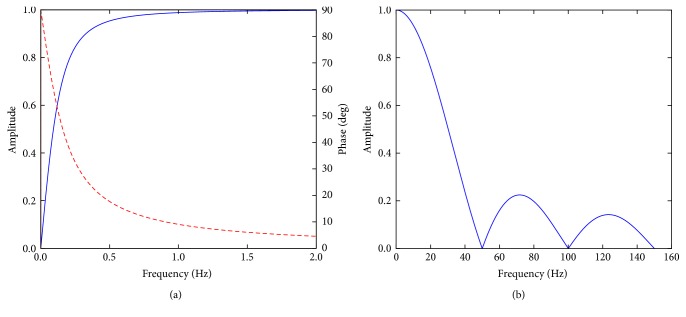
The (a) high-pass and (b) low-pass filters. The phase response for the high-pass filter is shown as a dashed red line, showing an increasing nonlinear effect at very low frequencies. The phase for the low pass is linear, such that all frequencies are delayed by the same time.

The high frequencies are removed by a low-pass filter with a cut-off frequency close to 22 Hz. This is achieved using a moving average filter, which is a simple implementation of an FIR filter. This filter has a linear phase so as not to distort the waveforms and a low roll-off rate. With careful implementation of the filter taps, this can also be designed to minimise multiples of 50 Hz noise (Figure 3) [18].

The high-pass filter is more complicated to design due to the very low (0.15 Hz) cut-off frequency. The primary purpose of this filter is to block the dominant DC background on which the PPG signal is superimposed. For speed of processing and response, we have adopted a first-order digital high-pass IIR filter with a cut-off of 0.15 Hz, and the transfer function is as follows:

(1) $$\begin{array}{c} H\left(\;z\;\right)=\frac{1-z^{-1}}{1-\alpha \cdot z^{-1}}\;\text{where }\alpha <1. \end{array}$$

Although this filter is an IIR filter and has a nonlinear phase response, this nonlinearity is concentrated at very low frequencies below the filter cut-off frequency. The filter responses are shown in Figure 3 and combined give a bandpass with the required attributes.

The filtered signal is then sliced into individual pulses. This could be done with the PPG traces, finding the troughs between the pulses; however, this can be problematic either with a weak signal or when there is substantial noise. A more reliable method is using the R-wave gating from an ECG signal. In this study, the R-waves from the ECG signal have been extracted using the method developed by Pan and Tompkins [19] and the troughs between the pulses found from the subsequent minima following each R-wave. Each resulting pulse then has a constant background removed and is normalised in amplitude and duration. As the clustering method is processor-intensive, there is a “sanity check” on the pulse to check that it is pulse-like in form. This is designed to be computationally fast and is used to discard obvious nonpulses. This check averages the amplitude of the samples in the first 5%, middle 90%, and last 5% of the pulse. The average of the middle section must be 1.5x larger than the biggest of the average of the first and the average of the last sections. This ensures that the pulse amplitude starts low, goes up, and then returns low, giving confidence that a periodic pulse-like feature is present for subsequent analysis.

### 2.3. Pulse Clustering

The algorithm saves the pulse into an array. This pulse is then compared to all previous pulses by comparing the amplitude of each sample within the pulse. In order to compare the pulses, distance metrics were tested, including calculating the Pearson correlation coefficient, the Kendall rank correlation coefficient, the Spearman rank correlation coefficient, and the root mean square error (RMSE). Each of these distance metrics is optimised differently; by using a subset of data and visual comparisons of the clusters, RMSE produced the most appropriate clustering. RMSE also has the advantage of being computationally simple and therefore fast.

Each pulse forms a new cluster and is the centre of that cluster. In addition, each pulse is placed into any other cluster, where the RMSE between this pulse and the pulse at the centre of that cluster is below a threshold value. In this way, N pulses create N clusters, each populated with pulses with an RMSE from the centre pulse less than a preset threshold.

### 2.4. Termination

After each pulse has been clustered, the number of pulses in each cluster is calculated. If any cluster has sufficient pulses for the algorithm requirements, then the loop is terminated, and an averaged (normalised) pulse is returned. As the pulses are normalised in time, the median pulse is calculated by finding the median of each point on the pulse. If there is no cluster with sufficient pulses, then the algorithm accepts more data, or if a predefined time-limit has been reached, then the algorithm terminates with a time-out error. This protects the algorithm from running continuously with no output.

### 2.5. Algorithm Validation

To validate the algorithm, a clean PPG signal was analysed using the filtering described above, however with the clustering turned off. This returned an averaged representative pulse shape. The signal was then digitally contaminated with noise, and the analysis was repeated with and without clustering. The output of these two methods of analysis was compared to the representative pulse from the clean signal.

## 3. Results and Discussion

**Figure 4:**
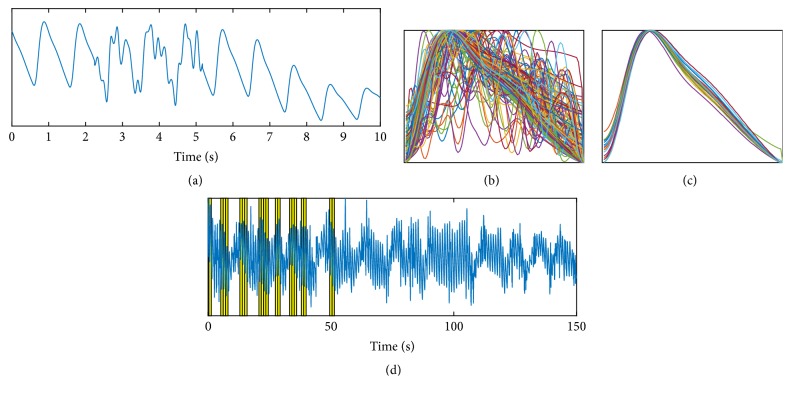
(a) PPG trace with artificial “movement” noise added. (b) and (c) show full pulse set and clustered pulses. (d) shows full trace with cluster pulses highlighted.

Figure 4(a) shows an extract from a PPG trace with artificial noise added to approximately 30% of the signal. The noise has been designed to replicate movement noise as seen in the middle trace in Figure 1.  Figure 4(b) shows all of the pulses (without clustering), and Figure 4(c) shows just those pulses within the largest cluster. The duration of the input PPG signal is 150 seconds; however, the algorithm with clustering self-terminates when any cluster contains 20 pulses.  Figure 4(d) shows the full trace of the PPG with the 20 pulses contributing to the largest cluster highlighted in yellow. Note that there are no pulses selected from the end of the trace as the algorithm is analysing the trace as if in real time and therefore terminates when there are sufficient (in this case 20) pulses.

**Figure 5:**
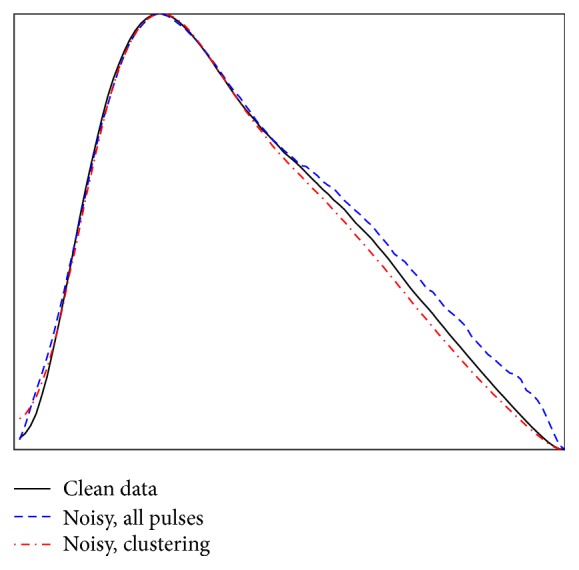
Median peaks from the clean data pulses, all pulses after noise is added, and the median from using clustering on the noisy data.

It is clear from Figure 4 that the clustering successfully selects pulses of a similar shape and these visually appear to be a physiologically representative set.  Figure 5 shows the median of all the pulses and the median from the cluster pulses together with the median of all the pulses from the original clean data. It can be seen in Figure 5 that the cluster set produces a median pulse much closer to the clean data median pulse than the median from all pulses.

**Figure 6:**
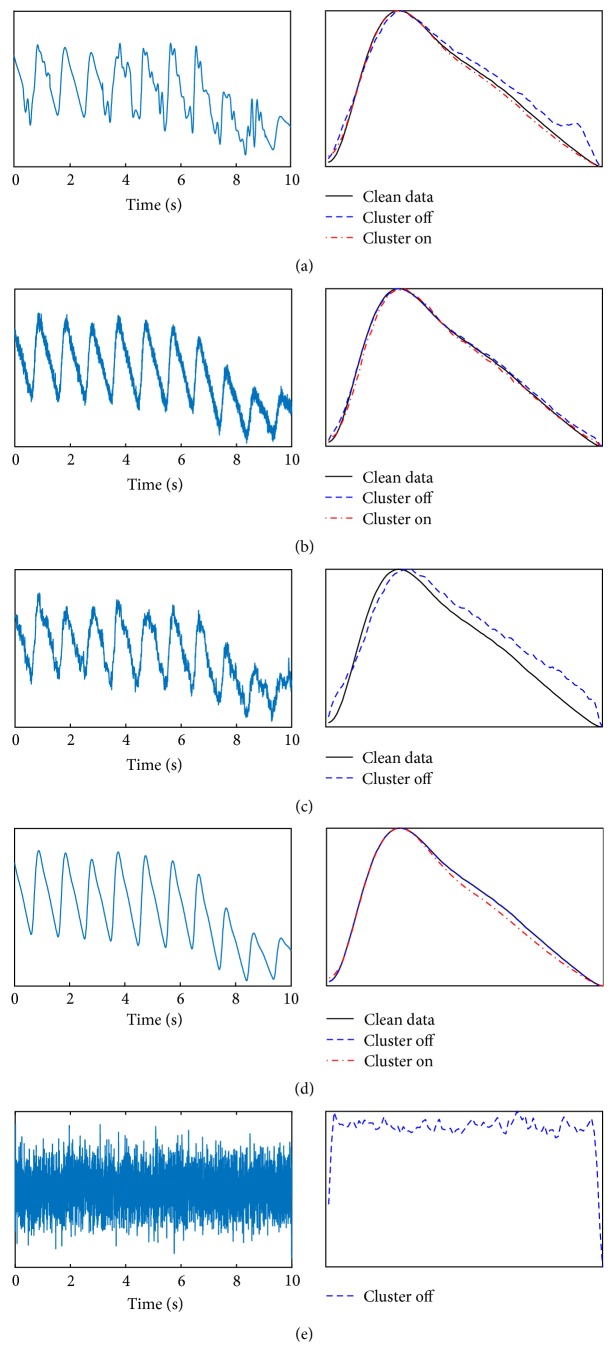
Comparison between clustering and nonclustering for a variety of inputs. (a) Simulated movement artefacts, (b) simulated electrical noise, (c) simulated electrical artefact and movement noise, (d) no noise, and (e) white noise as an input. Note that in (c) and (e) a cluster of sufficient size was not formed; therefore the algorithm reported no result.

In order to validate the algorithm, we have quantified the difference between the clean, cluster, and noncluster median pulses using the same RMSE comparison. We have simulated movement noise, constant electrical noise (similar to the lower trace in Figure 1), a combination of movement and electrical noise, no noise, and white noise with no PPG signal, all shown in Figure 6. The right-hand panels show a comparison between the median of the clean data, the median of the data with noise added, and the median of the cluster. In Figure 6(c), a combination of movement and electrical noise prevented a cluster forming within the defined thresholds, and the median of all of the noisy pulses is deviating significantly from the clean signal. In Figure 6(e), we used white noise as the input. Again, there was no cluster formed; however, the data was still gated by the ECG R-waves and therefore a median of pulses of noise is produced. The ability of the cluster algorithm to not produce a median pulse is extremely important as it prevents returning a false signal for disease diagnosis.

Table 1 shows the RMSE values for each of these cases, showing in all situations where a cluster was formed that this produced a median pulse closer to the median pulse from the original clean data. No RMSE values can be calculated for a white noise input as seen in Figure 6(e), as there is no original “clean” data signal. The biggest improvement of the cluster algorithm is seen with a high proportion of movement noise on the signal. The movement noise is sporadic and only affects individual pulses; removing these pulses can result in a dramatic improvement. By contrast, electric noise is applied to all pulses, and therefore it is more difficult for a cluster based algorithm to select individual pulses without noise. Despite this, with simulated electrical noise, the cluster analysis produced an RMSE almost half the size of the RMSE from a median of all the pulses.

**Figure 7:**
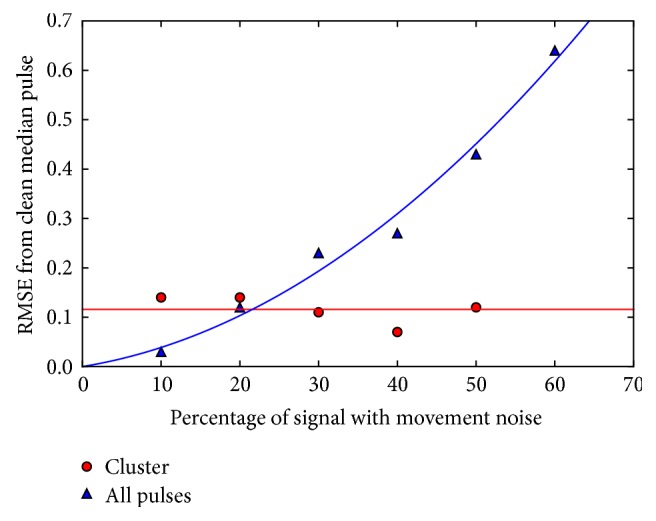
Comparison between the cluster and noncluster algorithms with increasing noise.

The performance of the cluster algorithm increased as the proportion of the signal contaminated by movement noise was set from 10% to 50%. The comparison between the RMSE of the cluster algorithm and a median of all pulses is shown in Figure 7. This shows that the cluster result returns broadly consistent results independent of the noise added to the signal, until it is no longer able to produce a result. By comparison, the median of all pulses becomes increasingly poor at representing the original signal. At very low noise levels, a better result can be achieved through averaging over more pulses; therefore the cluster algorithm limiting the number of pulses averaged to 20 performs worse. However, as the noise level increases, the clustering algorithm is superior. Note also that the time for the algorithm to return a result increases with the cluster method as the noise content increases, and indeed noise contamination greater than 50% could be achieved by increasing the time-out limit. By comparison, a median of all pulses will take a fixed duration of time independent of the signal quality and will therefore take significantly longer than the cluster method where there is low noise contamination. For real-world clinical application, the algorithm has the scope to indicate when a probe has become unattached from the measurement site or has failed.

Further work linked to photoplethysmography can include assessments of our method approach across a wide range of recordings from healthy subjects and vascular patients and also for different peripheral measurement sites such as the ear lobe and finger pads.

## 4. Conclusions

We have shown that clustering can be used within a real-time algorithm to minimise the effects of noise on a periodic physiological signal, with an algorithm that can be tailored to individual signal type. For this paper, we have explored its value for photoplethysmography waveforms as the input signal, where a dramatic reduction in the effect of noise on the output result has been demonstrated. Furthermore, if there is insufficient quality of data, the algorithm returns a null result rather than an incorrect median pulse. The algorithm returns a consistent result as the noise on the signal is increased and at low noise levels can produce a result quickly and efficiently. This algorithm was developed to be computationally fast, such that it could be run in real time on an embedded microcontroller within a portable medical device.

## Figures and Tables

**Table 1:** RMSE comparisons between noncluster and cluster algorithms for different noise situations. Note that where there is significant noise the cluster method returns a time-out error.

Panel in Figure 6	Simulated noise	RMSE between clean median pulse and median of all noisy pulses (153 pulses)	RMSE between clean median pulse and median of cluster (20 pulses)
	Movement artefact (10% noise)	0.03	0.14
	Movement artefact (20% noise)	0.12	0.14
	Movement artefact (30% noise)	0.23	0.11
	Movement artefact (40% noise)	0.27	0.07
(a)	Movement artefact (50% noise)	0.43	0.12
	Movement artefact (60% noise)	0.64	—
(b)	Electrical noise	0.12	0.09
(c)	Movement (30%) and electrical noise	0.66	—
